# Effects of SGLT2 Inhibitors with and without Metformin in High-Risk, Treatment-Naïve Patients with Diabetes

**DOI:** 10.3390/jcm13051387

**Published:** 2024-02-28

**Authors:** Chen-Yu Huang, Jen-Kuang Lee

**Affiliations:** 1 Cardiovascular Center, Cathay General Hospital, No. 280, Sec. 4, Ren’ai Road, Da’an District, Taipei City 106438, Taiwan; chenyo2007@gmail.com; 2 Division of Cardiology, Department of Internal Medicine, National Taiwan University Hospital, No. 7, Zhongshan S. Road, Zhongzheng District, Taipei City 100225, Taiwan; 3 Division of Cardiology, Department of Internal Medicine, National Taiwan University College of Medicine, Taipei City 100225, Taiwan; 4 Department of Laboratory Medicine, National Taiwan University College of Medicine and Hospital, Taipei City 100225, Taiwan; 5 Telehealth Center, National Taiwan University Hospital, Taipei City 100225, Taiwan; 6 Cardiovascular Center, National Taiwan University Hospital, Taipei City 100225, Taiwan

**Keywords:** sodium-glucose co-transporter-2 inhibitors, diabetes mellitus, atherosclerotic cardiovascular disease

## Abstract

**Background:** Contemporary evidence supports the cardiovascular and renal benefits of sodium-glucose co-transporter-2 inhibitors (SGLT2is) in patients with diabetes. While metformin has traditionally been recommended as a first-line treatment, its exact role in improving cardiovascular outcomes remains uncertain. This study aims to evaluate the impact of combination therapy with metformin on the cardiovascular and renal outcomes in high-risk, treatment-naïve diabetic patients who have undergone SGLT2i therapy. **Methods:** Using the National Health Insurance Research Database in Taiwan, a retrospective cohort study was conducted. Treatment-naïve patients with diabetes and established atherosclerotic cardiovascular disease (ASCVD) undertaking SGLT2i therapy from 1 January 2016 to 31 December 2021 were included. Patients were categorized based on the concomitant use of metformin. Propensity score matching was employed to minimize confounding factors. The primary outcome was major adverse cardiovascular events (MACEs), with secondary outcomes including cardiovascular death, hospitalization for heart failure, and renal outcomes. **Results:** In total, 10,151 treatment-naïve diabetic patients with ASCVD were identified, with 2570 in the only SGLT2i therapy group and 7581 in the SGLT2i plus metformin group. In total, 2262 pairs were analyzed after propensity score adjustment. The risk of MACEs (36.6 vs. 42.1 events per 1000 person-years; hazard ratio 0.87, 95% confidence interval 0.70–1.09) and other outcomes did not significantly differ between the two treatment groups. **Conclusions:** In high-risk, treatment-naïve diabetic patients, initiating SGLT2i therapy alone or in combination with metformin resulted in comparable cardiovascular and renal outcomes. These findings suggest that metformin might not be mandatory as a first-line treatment for achieving cardiovascular benefits in such patients.

## 1. Introduction

Sodium-glucose co-transporter-2 inhibitors (SGLT2is), a novel class of anti-diabetic agents, function by inhibiting renal glucose reabsorption through the blockade of SGLT2 cotransporters in the proximal tubules. This mechanism reverses glycemia and glomerular hyperfiltration, which are key to its renoprotective effects [1]. However, the exact mechanisms responsible for its cardioprotective effects remain unclear. It appears that SGLT2is enhance ketogenesis in cardiomyocytes [2], while also mitigating the sarcolemmal sodium and calcium overload condition in failing cardiomyocytes [3]. Recent investigations have revealed that SGLT2is improve myocardial energetics and ameliorate anemia by promoting erythropoiesis and iron metabolism [4,5,6]. Myocardial iron deficiency has been seen to contribute to advancing cardiac remodeling and impaired mitochondrial respiration [7]. Substantial evidence has confirmed the beneficial effects of SGLT2is in reducing major adverse cardiovascular events (MACEs), hospitalization for heart failure (HHF), and renal outcomes in patients with type 2 diabetes (T2DM) [8]. Dapagliflozin and empagliflozin have expanded their indications based on their efficacy in reducing MACEs, cardiovascular death, and HHF, as well as improving renal outcomes, even in the non-diabetic population, indicating that the cardioprotection benefits of SGLT2is were independent of their glucose-lowering properties [9,10]. Although metformin has traditionally been recommended as the first-line treatment for T2DM, its role is primarily based on its low cost, effects on body weight, and benefits demonstrated in the UK Prospective Diabetes Study (UKPDS) [11]. However, the UKPDS was conducted over two decades ago, prior to the widespread use of other cardioprotective therapies, making direct comparisons with contemporary trials of SGLT2is challenging. Meta-analyses have not provided clear evidence of the cardiovascular benefits of metformin for individuals with T2DM and there are limited data on its effects on renal outcomes [12,13].

Most of the contemporary cardiovascular and renal outcome trials of novel antidiabetic agents were conducted with the background use of metformin. Though subsequent subgroup analyses and meta-analysis have explored the potential impact of metformin on cardiovascular, renal, and mortality outcomes, the applicability of these findings is uncertain due to the limited number of randomized controlled trials (RCTs) focusing on concomitant metformin use [14,15]. It is important to note that subgroup analyses, while valuable, are not definitive and should undergo formal evaluation for credibility using the relevant RCTs. New guidelines from the European Society of Cardiology, in collaboration with the European Association for the Study of Diabetes, recommend the early use of SGLT2is in high-risk T2DM patients, regardless of whether they are treatment-naïve or not [16]. Most DM guidelines, including those within the American Diabetes Association (ADA)/European Association for the Study of Diabetes (EASD) consensus report, recommend that metformin should be used as a first-line treatment [17]. Therefore, utilizing the largest nationwide health insurance database in Taiwan, we conducted a retrospective cohort study to investigate the effects of SGLT2i therapy on cardiovascular, renal, and mortality outcomes in high-risk, treatment-naïve T2DM patients both with and without baseline metformin use.

## 2. Materials and Methods

### 2.1. Data Source

This study was designed as a retrospective cohort study which utilized data from the Taiwan National Health Insurance Research Database (NHIRD). The National Health Insurance (NHI) program was introduced in 1995 with the aim of providing comprehensive healthcare coverage to all residents of Taiwan. Currently, the NHI program extends to around 99.8% of the population, which amounts to approximately 24 million individuals. This database contains health insurance data obtained from beneficiaries, and access to these data is granted only after obtaining ethical approval. The NHIRD includes data on all patients covered by the NHI program and is managed by the Health and Welfare Data Science Center (HWDC). Ethical approval for this study was obtained from the institutional review board. The IRB determined that patient consent could be waived since the original identification numbers of the patients were encrypted in the NHIRD.

### 2.2. Study Population

The Taiwan NHI program initiated SGLT2i therapy reimbursement for treating diabetes since 1 January 2016 and we gathered all prescription information of available SGLT2i types, dosage, date of prescription, and duration of treatment from the electronic pharmacy prescription directory in the NHIRD from 1 January 2016 to 31 December 2021. Newly diagnosed T2DM patients were identified during the same interval using the International Classification of Diseases, 9th Revision, Clinical Modification (ICD-9-CM) diagnostic codes and were confirmed by prior electronic medical records indicating they had not received antidiabetic medication (including insulin), thus were regarded as part of the treatment-naïve T2DM population. Patients with atherosclerotic cardiovascular disease (ASCVD) was defined as those being documented with either one or a combination of the ICD-9-CM diagnostic codes of coronary artery disease, myocardial infarction, peripheral artery disease, or cerebrovascular accident (including any type of stroke). We verified the accuracy of the ASCVD status by reviewing ambulatory and inpatient claims data. The date of the first SGLT2i prescription was defined as the index date. Finally, newly diagnosed or treatment-naïve T2DM individuals with established ASCVD from 1 January 2016 to 31 December were selected for further analysis after excluding subjects who had missing demographics, were aged less than 20, or were followed for less than 90 days. The main exposure of interest was concomitant metformin use after the index date, information of which was extracted from the electronic pharmacy prescription directory in the NHIRD.

### 2.3. Covariate Measurements

The covariates in our study included age, sex, ASCVD characteristics, concurrent medication, and comorbid conditions. To ensure diagnostic accuracy, comorbidities were determined to have at least two outpatient diagnoses or any inpatient diagnosis prior to the index date, including atrial fibrillation, hypertension, dyslipidemia, chronic kidney disease (CKD), chronic obstructive pulmonary disease, and malignancy. Other historical events requiring hospitalization comprise heart failure, embolic events, and venous thromboembolism, which were defined to have inpatient diagnoses preceding the index date. Concomitant medications other than anti-diabetic drugs within three months before and after the index date were extracted, including anti-platelet agents, anti-coagulants, statins, anti-hypertensive agents, and other medications. The concurrent use of other classes of anti-diabetic medications (including dipeptidyl peptidase 4 inhibitor [DPP4i], glucagon-like peptide-1 receptor agonist [GLP-1RA], sulfonylurea, thiazolidinedione, alpha glucosidase inhibitors, glinide, and insulin) after the index date were also recorded.

### 2.4. Outcome Definitions

The primary outcome in our study was MACEs, comprising cardiovascular death, myocardial infarction, and ischemic stroke. The secondary outcome was the composite of cardiovascular death and HHF. Other outcomes examined included newly diagnosed chronic kidney disease (CKD) and all-cause death. The information of the date and causes of death were available by linking to the Taiwan Death Registry database in the HWDC. The occurrence of myocardial infarction, ischemic stroke, and HHF were verified by using the principal discharge diagnosis and inpatient claims data; newly diagnosed CKD was ascertained by two outpatient diagnoses or any single inpatient diagnosis. Individuals were followed from the index date to the date of outcome occurrence, death, three months follow-up after starting metformin use for the SGLT2i therapy alone group, or 31 December 2021, whichever came first.

### 2.5. Statistical Analysis

In order to address potential confounding factors when comparing outcomes between different groups, we utilized a propensity score matched cohort with the greedy algorithm. The propensity score, calculated through a multivariable logistic regression model without considering interaction effects among covariates, incorporated all the available covariates, including baseline characteristics, concurrent medication, and comorbid conditions (with the follow-up year replaced by the index date). Individuals in the SGLT2i therapy alone group and the SGLT2i therapy with metformin group were matched at a 1:1 ratio. The caliper was set as 0.2, the matching order was random, and replacement was not allowed. The quality of matching was assessed by the absolute value of standardized difference (STD) between the study groups, in which a value of less than 0.10 was considered negligible [18].

The risk of fatal outcomes (i.e., cardiovascular death, MACEs, all-cause death, and the composite of cardiovascular death and HHF) between groups was compared using the Cox proportional hazard model. The incidence of non-fatal outcomes between groups was compared using the Fine and Gray subdistribution hazard model which considered all-cause death a competing risk. Subgroup analyses were conducted on two main outcomes, including MACEs and the composite of cardiovascular death and HHF. The selected subgroup variables were age (<65 vs. ≥65 years), sex, each type of ASCVD, previous HHF, hypertension, dyslipidemia, CKD, and the concomitant use of statins. The study groups (SGLT2i therapy alone vs. SGLT2i therapy with metformin) were the only explanatory factor in the above survival analyses. A two-sided *p* value below 0.05 was considered statistically significant. All analyses were conducted using SAS version 9.4 (SAS Institute, Cary, NC, USA).

## 3. Results

### 3.1. Patient Inclusion

**Figure 1:**
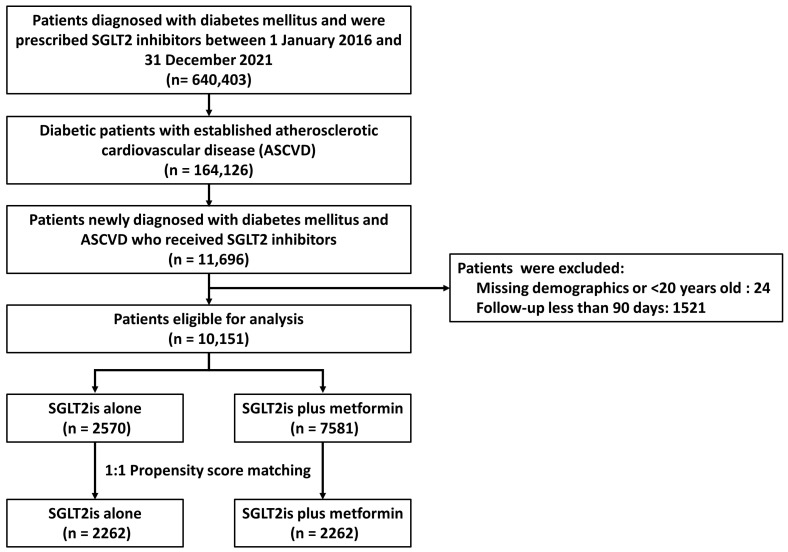
Flow diagram for the inclusion and exclusion of the study individuals.

A total of 10,151 treatment-naïve T2DM patients with established ASCVD were identified based on the inclusion criteria from 1 January 2016 to 31 December 2021. The study focused on two groups: those initially treated with SGLT2is alone (2570 patients) and those treated with a combination of SGLT2is and metformin (7581 patients). Prior to matching, the SGLT2is alone group was observed for an average of 1.8 years (standard deviation [SD] of 1.3 years), while the SGLT2is plus metformin group was observed for an average of 2.1 years (SD of 1.9 years). Following propensity score matching, both groups consisted of 2262 individuals. The study flowchart is illustrated in Figure 1.

### 3.2. Baseline Characteristics

The baseline characteristics of the entire and propensity score matched cohorts are demonstrated in Table 1. Among different ASCVD characteristics, coronary artery disease was dominant (83%), followed by myocardial infarction (27.5%), stroke (15.6%), and peripheral artery disease (7.9%). Before propensity score matching, the SGLT2is alone group was more elderly (64.9 vs. 58.5 years, STD 0.52); had a higher prevalence of atrial fibrillation, CKD, and previous HHF; and had a higher prescription rate of anticoagulation, diuretics, and spironolactone and a lower prescription rate of aspirin, dipeptidyl peptidase-4 inhibitor, sulfonylurea, thiazolidinedione, and insulin at baseline. After matching, all the baseline characteristics were well balanced between the groups, as demonstrated by all the STD values being <0.1.

### 3.3. MACEs and Other Outcomes

**Figure 2:**
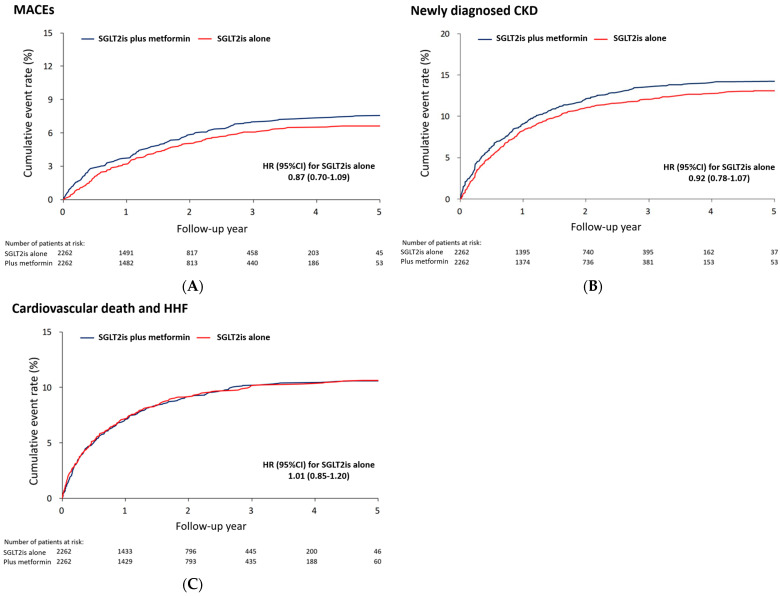
The cumulative event rates of MACEs (**A**), newly diagnosed chronic kidney disease (**B**), and the composite outcome of cardiovascular death and HHF (**C**) in treatment-naïve diabetic individuals receiving SGLT2is with and without concomitant use of metformin in the propensity score matched cohort. SGLT2is, sodium–glucose cotransporter 2 inhibitors; MACEs, major adverse cardiac events CKD, chronic kidney disease; HHF, hospitalization for heart failure; HR, hazard ratio; CI, confidence interval; SHR, subdistribution hazard ratio.

Over a mean follow-up period of 1.9 years, no significant difference in the risk of MACEs was observed between individuals treated with SGLT2is alone and those receiving a combination of SGLT2is and metformin (36.6 vs. 42.1 events per 1000 person-years; hazard ratio [HR] 0.87, 95% confidence interval [CI] 0.70–1.09]) (Table 2 and Figure 2A). There was no significant difference in the risk of cardiovascular death, myocardial infarction, or ischemic stroke between the two treatment groups. Additionally, there was no notable distinction in the risk of progression to renal insufficiency between the group treated solely with SGLT2is and the group receiving both SGLT2is and metformin (77.9 vs. 85.6 events per 1000 person-years; subdistribution HR [SHR] 0.92, 95% CI: 0.78–1.07) (Figure 2B). The risk of the composite of cardiovascular death and HHF did not considerably differ between the two treatment groups (Figure 2C). No significant differences were observed among the groups in terms of other outcomes.

### 3.4. Subgroup Analysis

**Figure 3:**
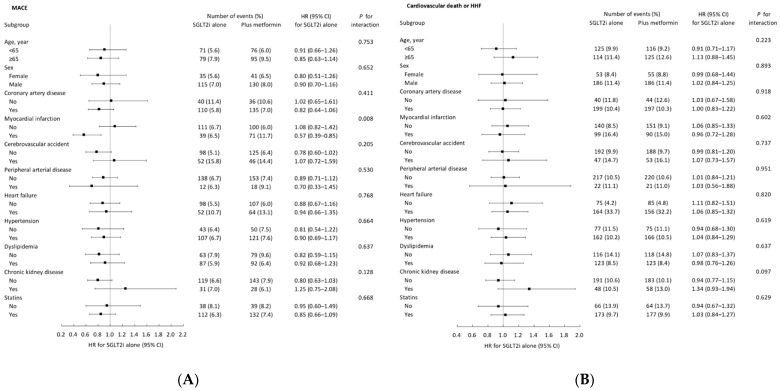
Subgroup analysis on outcomes of MACEs (**A**) and the composite outcome of cardiovascular death and HHF (**B**) using selected subgroup variables in the propensity score matched cohort. MACEs, major adverse cardiac events; HR, hazard ratio; CI, confidence interval; HHF, hospitalization for heart failure.

The effect of SGLT2is with or without metformin on MACEs was generally consistent across various subgroups, except for the individuals with a history of myocardial infarction (Figure 3A). In treatment-naïve individuals with type 2 diabetes, using SGLT2is without metformin was associated with a decreased risk of MACEs in those who had suffered previous events of myocardial infarction. (*p* for interaction = 0.008). In contrast, the impact of SGLT2 inhibitors, whether used alone or in combination with metformin, on the composite of cardiovascular death and HHF remained consistent across the various subgroups (Figure 3B).

## 4. Discussion

Our study represents the first real-world investigation comparing the effect of SGLT2is both with and without concomitant metformin in treatment-naïve T2DM patients with ASCVD. Specifically, we aimed to evaluate the occurrence of MACEs, renal outcomes, and other cardiovascular outcomes in a high-risk T2DM population. A substantial body of evidence has shown positive cardiovascular [19,20,21,22] and renal outcomes [23,24,25,26] associated with the use of SGLT2is in T2DM, although many of these studies primarily involved participants taking metformin at the baseline. This discrepancy in the evidence has led to debates regarding the initial treatment approach for high-risk T2DM patients. Through a comprehensive analysis of a nationwide retrospective cohort, we found that the use of SGLT2is alone resulted in a comparable risk of primary composite outcome (cardiovascular death, myocardial infarction, or ischemic stroke) when compared to the combination of SGLT2is and metformin as an initial medical treatment for treatment-naïve T2DM patients with ASCVD.

The cardioprotective effect of SGLT2is has been considered to be independent of its glucose-lowering properties. Recent evidence suggests that an increase in hematocrit, thereby enhancing the oxygen-carrying capacity, may contribute to this effect. Randomized evidence indicates that dapagliflozin administration in patients with T2DM leads to increased plasma erythropoietin and transferrin levels. This is accompanied by reduced serum hepcidin and ferritin concentrations, suggesting an enhanced iron utilization in hematopoiesis [4]. The post hoc analyses of randomized trials have revealed an elevation in the erythropoietin level, a decrease in hepcidin, and myocardial iron repletion following empagliflozin treatment in patients with heart failure [5,6]. Metformin (dimethylbiguanide), a biguanide anti-diabetic agent, has been commonly used and recommended as the first-line treatment in T2DM. Metformin modulates the glycometabolic control by activating the enzyme AMPK (AMP-activated protein kinase) in hepatocytes and skeletal muscle, thereby increasing glucose uptake and reducing gluconeogenesis and hepatic glycogenolysis [27]. It has also been administered for inducing ovulatory menstrual cycles in oligo-amenorrhoeic patients with polycystic ovary syndrome [28]. Despite being widely used, metformin has not been comprehensively investigated among high-risk T2DM individuals in outcome-based randomized trials. While conducting such trials could potentially alleviate uncertainties regarding the effectiveness of metformin in reducing MACEs, it would not provide insights into common therapeutic dilemmas with regards to the most suitable drugs or combinations, establishing the optimal sequence of drug application and identifying specific patient characteristics that would benefit from such treatment approaches.

The main evidence that supports metformin as the first-line treatment in T2DM in terms of reducing cardiovascular events comes from the UKPDS, which was conducted over two decades ago, prior to the extensive application of cardioprotective drugs including renin–angiotensin system inhibitors and statins [11,29]. As a newly emerging class of anti-diabetic drug, SGLT2is have been proven to provide both glycemic control and favorable cardiovascular and renal outcomes in T2DM [8]. In contrast, the impact of metformin on cardiovascular outcomes and mortality is still uncertain [30]. Most evidence in relation to the cardiovascular benefits of metformin were derived from studies involving relatively young, overweight patients with poorly controlled T2DM among North American and Northern European patients [31,32]. A previous meta-analysis suggested that metformin treatment in patients with T2DM was associated with a decreased cardiovascular risk, but none achieved statistical significance [12]. Nevertheless, another meta-analysis that evaluated the isolated effect of metformin compared to diet or a placebo did not show a cardiovascular benefit [33] and, in some cases, increased mortality in terms of intensive blood glucose control [34].

In our study, the characteristics of cohort and MACEs closely resembled those observed in prior clinical trials assessing cardiovascular outcomes associated with canagliflozin (2.69% per year), empagliflozin (3.74% per year), and ertugliflozin (3.9% per year) [19,21,22]. In our cohort, 83% of patients had coronary artery disease, 15.6% had cerebrovascular disease, and 7.9% had peripheral arterial disease. Additionally, 17% of patients had a history of heart failure. More than 80% of patients in our study were being treated with statins, with over half of them also receiving renin–angiotensin system blockade and β-blockers. A previous meta-analysis, conducted by Masson et al., examined the impact of SGLT2is on MACEs in metformin-free T2DM patients. The study found that while SGLT2is did not significantly reduce MACEs (OR: 0.85, 95% confidence interval: 0.63–1.15), it was associated with a notable decrease in HHF and cardiovascular death (OR: 0.67, 95% CI: 0.47–0.95) [14]. In contrast, our study demonstrated that treatment with SGLT2is alone resulted in a similar risk of MACEs (36.6 vs. 42.1 events per 1000 person-years; SHR 0.87, 95% CI: 0.70–1.09), HHF (53.1 vs. 50.8 events per 1000 person-years; SHR 1.06, 95% CI: 0.88–1.27), and cardiovascular death (15.6 vs. 16.3 events per 1000 person-years; SHR 0.96, 95% CI: 0.69–1.35) compared to the combination of SGLT2is and metformin.

Furthermore, another meta-analysis including six event-driven, randomized, placebo-controlled SGLT2i trials conducted by Neuen et al. demonstrated consistent and statistically significant relative risk reductions for all outcomes, regardless of metformin use at baseline [15]. In their report, it was shown that the use of empagliflozin without metformin had a stronger impact on reducing MACEs (HR 0.72; 95% CI 0.56–0.94) compared to the combination of empagliflozin and metformin (HR 0.92; 95% CI 0.77–1.10). Similar findings were observed in the CANVAS study, where canagliflozin without metformin resulted in a significant 24% reduction in MACEs (HR 0.76; 95% CI 0.61–0.94), whereas the addition of canagliflozin to existing metformin treatment led to a nonsignificant 9% reduction (HR 0.91; 95% CI 0.77–1.06). However, the subgroup analysis of the VERTIS-CV study revealed a comparable impact of ertugliflozin on MACEs, HHF, and cardiovascular death regardless of concurrent metformin usage [15]. While we lack a clear explanation for the discrepant results between our study and others, differences do exist among these studies, which might account for the variations in the outcomes. Notably, the prevalence of heart failure in the VERTIS-CV trial is 24%, higher than in our study (17%) and the EMPA-REG trial (10%). Additionally, the VERTIS-CV trial predominantly enrolled patients from Europe and North America, whereas the EMPA-REG study included more patients from Asia and South America [19,22]. Our study, on the other hand, solely involved an Asian population with T2DM.

The joint recommendations from the American Diabetes Association (ADA) and the European Association for the Study of Diabetes (EASD) suggest metformin as the first-line drug in glycemic management and the use of GLP-1 RA and SGLT2is with proven benefits in high-risk patients [17]. In patients with comorbid heart failure or CKD, the ADA/EASD guidelines suggest combining it with SGLT2is. Conversely, in T2DM patients with high cardiovascular risk, the ADA/EASD guidelines recommend adding SGLT2is only if GLP-1RA is contra-indicated or unavailable. However, the European guidelines from the European Society of Cardiology (ESC) recommend that SGLT2is and GLP-1RA may be initiated without metformin in T2DM with ASCVD or high cardiovascular risk [16]. This perspective implied that while metformin should be considered, it is not obligatory as the initial treatment for high-risk T2DM patients. Indeed, starting metformin in such patients should not prevent or postpone the initiation of evidence-supported SGLT2is or GLP-1RA. This distinct ESC treatment algorithm is based on the interpretation of predominantly favorable outcomes from both SGLT2is and GLP-1RA, independent of their glucose-lowering properties. Hence, prioritizing cardiovascular and renal protection over the sole focus on glycemic control via adopting a patient-centered approach should be considered in the future treatment paradigm of T2DM.

### Strengths and Limitations

Our study has notable strengths that deserve recognition. It employed a population-based, nationwide design, encompassing real-world patients with T2DM and established ASCVD. All comorbidities and medications were accurately documented, adhering to national health insurance regulations. The incidence of missing data related to patient withdrawal or loss to follow-up was minimal due to the mandatory documentation and coding system mandated by national insurance regulations. To the best of our knowledge, this is the largest Asian nationwide registry to date, focusing on a high-risk T2DM population, examining the effect of SGLT2is in reducing cardiovascular, renal, and mortality outcomes, regardless of background metformin use.

However, it is important to consider several factors when interpreting the results of our study. Firstly, our study design was not prospective or randomized. Although we employed propensity score matching to minimize differences between groups, there may still be biases from unmeasured confounding factors that were not accounted for. Secondly, our analysis was limited by the use of claims data, which means we were unable to provide detailed information on laboratory parameters such as serum glucose level, HbA1c, serum creatinine, albuminuria, or LDL-cholesterol level, all of which can influence cardiovascular and renal prognosis. This limitation is a significant drawback of our current analysis. Thirdly, our study exclusively focused on an Asian population, and it remains uncertain whether the findings can be generalized to other ethnic groups. Lastly, we included treatment-naïve T2DM patients with established ASCVD, which implies that some individuals might have an unknown duration of unrecognized T2DM without any prior treatment. Further studies with longer follow-up periods and larger patient cohorts are necessary to confirm or refute our findings.

## 5. Conclusions

Among individuals with T2DM and ASCVD who had not received prior treatment, our study found that using SGLT2is alone or in combination with metformin resulted in comparable risks of various outcomes. These findings suggest that initiating evidence-supported SGLT2is alone could be as effective as combining SGLT2is with metformin in high-risk, treatment-naïve patients with T2DM, in terms of cardiovascular benefits.

## Figures and Tables

**Table 1:** Baseline characteristics of diabetic patients with established ASCVD who received SGLT2is with and without concomitant use of metformin.

		Before Matching			After Matching		
Variable	Total (*n* = 10,151)	SGLT2is Alone (*n* = 2570)	Plus Metformin (*n* = 7581)	STD	SGLT2is Alone (*n* = 2262)	Plus Metformin (*n* = 2262)	STD
Age, year	60.1 ± 12.4	64.9 ± 13.0	58.5 ± 11.8	0.52	63.0 ± 12.4	63.1 ± 11.7	−0.01
Male, sex	7454 (73.4)	1840 (71.6)	5614 (74.1)	−0.06	1638 (72.4)	1629 (72.0)	0.01
ASCVD							
Peripheral artery disease	798 (7.9)	235 (9.1)	563 (7.4)	0.06	191 (8.4)	199 (8.8)	−0.01
Coronary artery disease	8424 (83.0)	2166 (84.3)	6258 (82.5)	0.05	1912 (84.5)	1923 (85.0)	−0.01
Myocardial infarction	2789 (27.5)	674 (26.2)	2115 (27.9)	−0.04	599 (26.5)	605 (26.7)	−0.01
Cerebrovascular accident	1584 (15.6)	402 (15.6)	1182 (15.6)	0.00	329 (14.5)	319 (14.1)	0.01
Comorbidity							
Atrial fibrillation	767 (7.6)	342 (13.3)	425 (5.6)	0.27	220 (9.7)	219 (9.7)	0.00
Hypertension	7032 (69.3)	1813 (70.5)	5219 (68.8)	0.04	1588 (70.2)	1593 (70.4)	0.00
Dyslipidemia	6593 (64.9)	1612 (62.7)	4981 (65.7)	−0.06	1466 (64.8)	1441 (63.7)	0.02
Chronic kidney disease	1721 (17.0)	597 (23.2)	1124 (14.8)	0.22	446 (19.7)	459 (20.3)	−0.01
COPD	757 (7.5)	261 (10.2)	496 (6.5)	0.13	211 (9.3)	206 (9.1)	0.01
Malignancy	540 (5.3)	191 (7.4)	349 (4.6)	0.12	148 (6.5)	150 (6.6)	0.00
History of event							
Heart failure	1727 (17.0)	673 (26.2)	1054 (13.9)	0.31	484 (21.4)	487 (21.5)	0.00
Embolic event	120 (1.2)	36 (1.4)	84 (1.1)	0.03	27 (1.2)	32 (1.4)	−0.02
Venous thromboembolism	76 (0.7)	25 (1.0)	51 (0.7)	0.03	20 (0.9)	23 (1.0)	−0.01
Concomitant medications							
Anti-platelet agents							
Aspirin	6378 (62.8)	1326 (51.6)	5052 (66.6)	−0.31	1253 (55.4)	1278 (56.5)	−0.02
Clopidogrel/Ticagrelor/Prasugrel	3495 (34.4)	884 (34.4)	2611 (34.4)	0.00	763 (33.7)	763 (33.7)	0.00
Anti-coagulants	864 (8.5)	371 (14.4)	493 (6.5)	0.26	244 (10.8)	233 (10.3)	0.02
Anti-diabetic medications							
DPP4i	1382 (13.6)	114 (4.4)	1268 (16.7)	−0.41	114 (5.0)	112 (5.0)	0.00
GLP1RA	21 (0.2)	4 (0.2)	17 (0.2)	−0.02	4 (0.2)	5 (0.2)	−0.01
Sulfonylurea	1933 (19.0)	89 (3.5)	1844 (24.3)	−0.63	89 (3.9)	93 (4.1)	−0.01
Thiazolidinedione	568 (5.6)	20 (0.8)	548 (7.2)	−0.33	20 (0.9)	22 (1.0)	−0.01
Alpha glucosidase inhibitors	269 (2.6)	33 (1.3)	236 (3.1)	−0.12	31 (1.4)	36 (1.6)	−0.02
Glinide	102 (1.0)	10 (0.4)	92 (1.2)	−0.09	10 (0.4)	12 (0.5)	−0.01
Insulin	598 (5.9)	35 (1.4)	563 (7.4)	−0.30	34 (1.5)	32 (1.4)	0.01
Other medications							
ACEi or ARBs	6999 (68.9)	1844 (71.8)	5155 (68.0)	0.08	1607 (71.0)	1599 (70.7)	0.01
Beta-blockers	5903 (58.2)	1525 (59.3)	4378 (57.7)	0.03	1315 (58.1)	1319 (58.3)	0.00
DCCBs	3239 (31.9)	848 (33.0)	2391 (31.5)	0.03	752 (33.2)	767 (33.9)	−0.01
Statins	8210 (80.9)	1984 (77.2)	6226 (82.1)	−0.12	1793 (79.3)	1787 (79.0)	0.01
NSAIDs/Cox-2	4961 (48.9)	1239 (48.2)	3722 (49.1)	−0.02	1096 (48.5)	1098 (48.5)	0.00
Diuretics	1788 (17.6)	685 (26.7)	1103 (14.5)	0.30	491 (21.7)	489 (21.6)	0.00
Spironolactone	1607 (15.8)	640 (24.9)	967 (12.8)	0.31	455 (20.1)	459 (20.3)	0.00
Follow up year	2.0 ± 1.3	1.8 ± 1.3	2.1 ± 1.3	−0.24	1.9 ± 1.3	1.9 ± 1.3	−0.01

Abbreviations: ASCVD, atherosclerotic cardiovascular disease; SGLT2is, sodium–glucose cotransporter 2 inhibitors; COPD, chronic obstructive pulmonary disease; DPP4i, dipeptidyl peptidase 4 inhibitor; GLP1RA, glucagon-like peptide-1 receptor agonist; ACEi, angiotensin converting enzyme inhibitor; ARBs, angiotensin receptor blockers; DCCBs, dihydropyrinde calcium channel blockers; NSAIDs, non-steroidal anti-inflammatory drugs; Cox-2, cyclo-oxygenase-2 inhibitor; STD, standardized difference; Data are presented as percentage or mean ± standard deviation.

**Table 2:** Follow-up outcomes of diabetic patients with established ASCVD who received SGLT2is with and without concomitant use of metformin in the propensity score matched cohort.

	SGLT2is Alone		SGLT2is with Metformin			
Outcome	Number of Events (%)	Incidence (95% CI) *	Number of Events (%)	Incidence (95% CI) *	HR/SHR (95% CI) for SGLT2is Alone	*p* Value
Cardiovascular outcome						
Cardiovascular death	66 (2.9)	15.6 (11.9–19.4)	69 (3.1)	16.3 (12.4–20.1)	0.96 (0.69–1.35)	0.828
Ischemic stroke	68 (3.0)	16.4 (12.5–20.4)	81 (3.6)	19.7 (15.4–24.0)	0.84 (0.61–1.16)	0.290
Acute myocardial infarction	32 (1.4)	7.7 (5.0–10.3)	44 (1.9)	10.5 (7.4–13.6)	0.73 (0.46–1.15)	0.176
Composite MACEs	150 (6.6)	36.6 (30.7–42.4)	171 (7.6)	42.1 (35.8–48.4)	0.87 (0.70–1.09)	0.222
Other outcomes						
Newly diagnosed CKD	296 (13.1)	77.9 (69.0–86.8)	323 (14.3)	85.6 (76.2–94.9)	0.92 (0.78–1.07)	0.261
All-cause death	94 (4.2)	22.3 (17.8–26.8)	114 (5.0)	26.9 (22.0–31.9)	0.83 (0.63–1.09)	0.177
Hospitalization for heart failure	212 (9.4)	53.1 (46.0–60.3)	202 (8.9)	50.8 (44.0–57.8)	1.06 (0.88–1.27)	0.572
CV death and HHF	241 (10.7)	60.4 (52.8–68.0)	239 (10.6)	60.1 (52.5–67.7)	1.01 (0.85–1.20)	0.918

Abbreviations: ASCVD, atherosclerotic cardiovascular disease; SGLT2is, sodium–glucose cotransporter 2 inhibitors; MACEs, major adverse cardiac events; CKD, chronic kidney disease; CV, cardiovascular; HHF, hospitalization for heart failure; CI, confidence interval; HR, hazard ratio; SHR, subdistribution hazard ratio; * Number of events per 1000 person-years.

## Data Availability

All data generated or analyzed during this study will be included in a future article. Further enquiries can be directed to the corresponding author.
